# A European Perspective on Auditory Processing Disorder-Current Knowledge and Future Research Focus

**DOI:** 10.3389/fneur.2017.00622

**Published:** 2017-11-21

**Authors:** Vasiliki (Vivian) Iliadou, Martin Ptok, Helen Grech, Ellen Raben Pedersen, André Brechmann, Naïma Deggouj, Christiane Kiese-Himmel, Mariola Śliwińska-Kowalska, Andreas Nickisch, Laurent Demanez, Evelyne Veuillet, Hung Thai-Van, Tony Sirimanna, Marina Callimachou, Rosamaria Santarelli, Sandra Kuske, Jose Barajas, Mladen Hedjever, Ozlem Konukseven, Dorothy Veraguth, Tone Stokkereit Mattsson, Jorge Humberto Martins, Doris-Eva Bamiou

**Affiliations:** ^1^Neuroscience, Medical School, Aristotle University of Thessaloniki, Thessaloniki, Greece; ^2^Department of Phoniatrics and Pediatric Audiology, Hannover, Germany; ^3^University of Malta, Msida, Malta; ^4^The Maersk Mc-Kinney Moller Institute, University of Southern Denmark, Odense, Denmark; ^5^Leibniz Institute for Neurobiology, Magdeburg, Germany; ^6^Audio-Phonological Center, St Luc’s University Hospital, Université Catholique de Louvain (UcL), Brussels, Belgium; ^7^Phoniatric and Pediatric Audiological Psychology, University Medical Center Göttingen, Georg-August-University, Göttingen, Germany; ^8^Department of Audiology and Phoniatrics, Nofer Institute of Occupational Medicine, Lodz, Poland; ^9^Department of Hearing-Language-Cochlear Implants, Kbo-Kinderzentrum München, Munich, Germany; ^10^ENT Department, CHU Liège, Liege, Belgium; ^11^CRNL and the Hospices Civils de Lyon, Lyon, France; ^12^Department of Audiology and Audiological Medicine, Great Ormond Street Hospital, London, United Kingdom; ^13^ENT and Audiology Department, Nicosia General Hospital, Nicosia, Cyprus; ^14^Department of Neurosciences, University of Padova, Padova, Italy; ^15^Latvia Children Hearing Center, Riga, Latvia; ^16^Clnica Barajas, Santa Cruz de Tenerife, Canary Islands, Spain; ^17^Faculty of Education and Rehabilitation Sciences, Speech Therapy Department, University of Zagreb, Zagreb, Croatia; ^18^Faculty of Health Sciences, Audiology Department, Istanbul Aydın University, Istanbul, Turkey; ^19^Department of Otorhinolaryngology, Head and Neck Surgery, University Hospital Zurich, University of Zurich, Switzerland; ^20^Faculty of Medicine and Health Sciences, Department of Neuromedicine and Movement Science, NTNU, Trondheim, Norway; ^21^Cochlear Implant Unit, Department of Otorhinolaryngology and Head and Neck Surgery, Centro Hospitalar e Universitário de Coimbra, Coimbra, Portugal; ^22^Faculty of Brain Sciences, UCL Ear Institute, University College London, London, United Kingdom

**Keywords:** auditory processing disorder, auditory processing, hearing, listening difficulties, ear, central auditory nervous system, hidden hearing loss, psychoacoustic

## Abstract

Current notions of “hearing impairment,” as reflected in clinical audiological practice, do not acknowledge the needs of individuals who have normal hearing pure tone sensitivity but who experience auditory processing difficulties in everyday life that are indexed by reduced performance in other more sophisticated audiometric tests such as speech audiometry in noise or complex non-speech sound perception. This disorder, defined as “Auditory Processing Disorder” (APD) or “Central Auditory Processing Disorder” is classified in the current tenth version of the International Classification of diseases as H93.25 and in the forthcoming beta eleventh version. APDs may have detrimental effects on the affected individual, with low esteem, anxiety, and depression, and symptoms may remain into adulthood. These disorders may interfere with learning *per se* and with communication, social, emotional, and academic-work aspects of life. The objective of the present paper is to define a baseline European APD consensus formulated by experienced clinicians and researchers in this specific field of human auditory science. A secondary aim is to identify issues that future research needs to address in order to further clarify the nature of APD and thus assist in optimum diagnosis and evidence-based management. This European consensus presents the main symptoms, conditions, and specific medical history elements that should lead to auditory processing evaluation. Consensus on definition of the disorder, optimum diagnostic pathway, and appropriate management are highlighted alongside a perspective on future research focus.

## Introduction

Hearing loss (HL), i.e., reduced pure tone sensitivity affects over 5% of the world’s population (1) and is the fifth leading cause of Years Lived with Disability, a component of the Disability-Adjusted Life Year, used to measure the global burden of disease (2). This hearing impairment, however, does not include the children and adult individuals who have normal hearing sensitivity but who experience auditory processing difficulties in everyday life that are reflected in reduced performance in other audiometric tests such as speech in noise or complex non-speech sound perception (3). This disorder is defined as “Auditory Processing Disorder” (APD) or “Central Auditory Processing Disorder” (CAPD) and is currently classified in ICD-10 as H93.25 for both acquired and congenital forms.^1^ It has been proposed that this disorder may be differentiated as (i) developmental APD, (ii) acquired APD (e.g., as a consequence of infections, neurologic trauma, stroke, or excessive noise exposure), and (iii) secondary APD (4). However, this categorization may be problematic in that it does not include presentations like “central presbycusis” (5) which may affect older adults with or without other cognitive impairments (6), or the distinct non-verbal processing disorders that are present in some but not all types of dementia (7, 8) of which few have a genetic component, or in neurological disorders with a genetic basis such as multiple sclerosis or in psychiatric disorders where auditory processing deficits are successfully addressed with auditory training (9). The ICD-11 Beta version includes APD under diseases of the ear following a proposal by two of the authors of this paper [Vasiliki (Vivian) Iliadou and Doris-Eva Bamiou] seconded/commented upon by other co-authors. The HL sequelae are well established, with higher rate of unemployment or employment at a lower grade of the hearing impaired (10) and increased risk for dementia (11), mental illness/depression (12, 13), and social isolation (14). APD may have similar detrimental effects on the affected individual, with low esteem/anxiety (15), anxiety, and depression (16) and symptoms in developmental APD, which may persist in adulthood (17). These may burden community inclusion while interfering with communicational, social, emotional, and academic-work aspects of life. Academic skills affected are mostly in higher-order language like reading and spelling (18). External factors contributing to negative psychosocial well-being in children with APD are environmentally based issues and support dissatisfaction (19).

Although APD is attracting increasing interest and recognition as a clinical entity among clinicians on the field and scientific organizations throughout the world [e.g., Ref. (4, 20–27)], there is ongoing debate regarding its diagnosis and management. Most of this debate is based on (i) rejecting currently used diagnostic Auditory processing test batteries even though they are the best available as a gold standard approach, (ii) reaching conclusions regarding APD based on research of APD suspected individuals who have a primary diagnosis of another developmental disorder, without explicitly testing for APD. As a consequence, there is limited availability of APD testing for the affected individuals both in Europe (28, 29) and beyond (30), while the expertise regarding APD within clinical audiological setups is variable.

The objective of the present paper is to define a baseline European APD consensus by experienced clinicians and researchers in this specific field of human auditory science. A second aim is to identify issues that future research needs to address in order to further clarify the nature of APD and thus assist in optimum diagnosis and evidence-based management. Authors of this position paper work in European countries and conduct both clinical and research work in the APD field. Five of the authors (Doris-Eva Bamiou, Martin Ptok, Vasiliki (Vivian) Iliadou, Christiane Kiese-Himmel, and Andreas Nickisch) of this position paper are at the top five publishing APD research while working exclusively in Europe according to the scopus database (United Kingdom, Germany, and Greece).

## When to Initiate APD Diagnostic

By the end of the nineteenth century, tuning forks and, some decades later, audiometric devices greatly helped to understand the extent and the site of lesion of HLs. Examinations focusing on pure tones thoroughly characterized and quantified threshold shifts which in most cases were congruent with impaired hearing of other acoustic signals in everyday life like spoken speech. However, clinical observations, especially in children and neurological adults, cast doubt that measurable threshold shifts could always explain the extent of reported hearing and listening impairment. Especially in cases with no threshold shift in pure tone audiometry but with hearing and listening difficulties, such incongruencies had to be explained. This finally led to the concept of APD in the 1950s (31), proposing a “more central” site of a lesion or deficit. It appears to be accepted by APD clinicians and researchers worldwide that there are no core symptoms unequivocally and specifically indicative for APD. Since many APD test procedures have been put forward, it is mandatory to decide which children/adults should be referred for further audiometric evaluation beyond conventional pure tone audiometry, speech audiometry in quiet, and objective electrophysiological/electroacoustic hearing tests. The European study group agrees that the following reported symptoms which cannot be explained otherwise should give reason to initiate an APD diagnostic assessment:

- difficulties understanding speech in complex listening situations (e.g., with background noise, when speech quality is degraded),
- being easily distracted, having difficulties to repeat or recall similar sounding words,
- having difficulties with sound localization and separation of auditory foreground from auditory background,
- exhibiting hyperacusis,
- seeking visual/facial cues to better understand,
- responding inadaequately,
- having disproportionate language acquisition problems or specific language impairment (refractory to language intervention procedures),
- exhibiting educational difficulties in the presence of a normal audiogram.

In addition, children with a history of chronic otitis media with effusion or recurrent upper respiratory tract infections should be examined for their auditory processing skills, particularly if there is a documented speech delay, phonological deficit, or communication issue. Other conditions like autism, attention deficit hyperactivity disorder, written language difficulties, deficits in executive functions, and dyslexia should call for APD diagnostic assessment too, especially in cases where an appropriate therapy by other professionals and educational input does not show expected or adequate results. Initiating the diagnostic evaluation for APD does not predict an APD diagnosis, since it is possible that children with behavioral indicators of APD show auditory processing skills within normal limits. This statement posits that children with these symptoms or disorders need and should be appropriately evaluated.

## APD Definition

### Current Knowledge

Auditory processing disorder is defined as a specific deficit in the processing of auditory information along the central auditory nervous system, including bottom-up and top-down neural connectivity (20, 23). Hearing sensitivity is in the majority of cases normal as measured by the pure tone audiometry. The deficits are thought to be infrequently associated with a macroscopic structural brain lesion identifiable by brain imaging at least in the pediatric population. However, there are pediatric cases with APD with established subtle structural abnormalities of the central auditory pathway in the presence or absence of other developmental disorders [e.g., Ref. (32–35)]. Atypical auditory processing may also be reflected in abnormal Auditory Brainstem Responses (ABR) recording in a limited number of cases, suggesting neural conduction deficits beyond the auditory nerve level (36). Auditory processing together with but beyond the early stages of cochlear amplification and auditory nerve transmission will impact on auditory perception of speech and of other complex auditory stimuli (37). Perception of such stimuli is usually not assessed by classical audiological evaluation. Thus assessing both audibility and perception of sounds with baseline audiometric tests [audiometry, Otoacoustic Emissions (OAE), ABR] in conjunction with central auditory processing tests provides a more ecological approach to auditory perception and hearing in everyday life.

### APD versus Hidden Hearing Loss (HHL) and Auditory Neuropathy Spectrum Disorders (ANSD)

The term “hidden hearing loss,” or supraliminal hearing disorders describe disorders that concern more temporal aspects of hearing impacting on the intelligibility of degraded speech by noise, reverberations, speed, limited articulation, or the localization of sounds sources rather than pure tone audiometric thresholds (38). It arises due to pathologies between the inner hair cells and auditory nerve fibers entry to the brainstem. There are also cases of progressive auditory neuropathies (e.g., in the presence of genetic or other peripheral neuropathies) that first present with auditory perceptual and processing deficits before the disease evolves and affects pure tone sensitivity (39). Conversely, there are cases diagnosed with ANSD at the time of neonatal hearing screening in whom the ABR normalize later on (40) and the audiological and clinical profile fulfils APD rather than ANSD criteria. This consensus acknowledges the overlap between APD, HHL, and ANSD, which may not always be easy to resolve with current audiological batteries; however, clinicians should attempt to localize the auditory deficit within the auditory nervous system as best as they can. It should be noted, however, that the majority of the authors of this position statement prefer the term APD; thus favoring the inclusion of individuals having peripheral mis-analysis of the acoustic cues with a secondary impact on the central auditory pathway functioning.

### What We Need to Decide On and Focus On Future Research

Defining deficits of auditory processing as “Central Auditory Processing Disorder” (CAPD) may exclude individuals whose problem starts at the level of the cochlea (HHL) but is not evident in the majority of classical audiological tests (OAE may be partially present) or cases of ANSD. In the latter deficits, usually present in speech perception and temporal resolution in association, in the majority of cases, with raised audiometric thresholds and ABR findings indicative of low-level involvement of the auditory pathway. If these clinical entities are to be included in one general disorder category then the term APD is more optimal, since processing abnormalities will be detected in all; however, the level of the pathology will be very broad and the natural course of the disease highly variable. In case we decide on a narrower disorder category that excludes inner ear and auditory nerve disorders, the term CAPD is best. There is, however, a need to consider the possibility of “mixed” presentations such as cochlear type HL and CAPD [e.g., Ref. (41)]. Defining normal hearing on the basis of a normal pure tone audiogram is at best insufficient (42). Auditory processing may start at the level of the cochlea and detailed evaluation of OAE should be incorporated with tests assessing neural function (ABR) and with results of specific diagnostic auditory processing tests (37).

Otitis media with effusion in its recurrent form (chronic) or other possible conditions of acoustic deprivation should be researched in terms of resulting transient HL and subsequent APD in longitudinal studies with specific diagnostic audiological tests.

## Diagnostic Pathway

### Current Knowledge

Auditory processing evaluation in the clinical setting is largely based on psychoacoustic test batteries of verbal and non-verbal stimuli (42–50) and may be ancillary completed with electrophysiological or objective audiological measures, such as acoustic reflex thresholds, tympanometry, ABR (speech and noise ABR included), or OAEs (suppression included). APD diagnosis (Table 1) is a highly demanding time consuming procedure with multidisciplinary information on the client’s profile being essential to test selection. Test results interpretation is facilitated by carefully evaluating an individual’s behavior as measured by specific questionnaires (51) and detailed clinical interview during history taking that includes an audiological history (of auditory symptoms, early development of auditory behaviors, and APD risk factors including a family history of APD as well as of HL). When choosing the auditory processing tests, language and cognitive confounds should be minimized (52). In this respect, a detailed receptive and expressive language evaluation may in some cases be necessary to choose those language-based audiologic tests which are appropriate regarding the individual’s language development, and, second, to identify language development impairments as possibly higher level disorder which may impact audiological results. Even though it should be kept in mind that the link between language, cognition, and auditory processing is complex with one process influencing another and *vice versa*, it is generally advised to adopt a multidisciplinary approach (when possible) to make sure that individual audiological findings are not primarily the result of a higher level disorder. History elements concerning symptoms, specific difficulties, onset of disorder, and musical training are taken into account to conclude whether an individual has APD or a comorbid challenge.

**Table 1:** Diagnostic criteria for auditory processing disorder (APD).

Diagnostic criteria for APD		
Criterion	Explained	Comments
Pure tone audiometry	Hearing sensitivity threshold ≦15 dB hearing loss for each frequency between 250 and 8,000 Hz in both ears (not average)	APD may be present in the presence of abnormal audiometric thresholds. However, APD diagnosis in the presence of raised audiometric thresholds may posit challenges and should only be made on the basis of validated tests that have been shown to be suprathreshold or have normative data that control for the level of audiometric loss

Abnormal auditory processing results	Performance at or below 2 SD below the mean in at least 2 validated auditory processing tests that assess different processes in at least one ear, including non-speech sounds	This does not incorporate notions of relative weakness in AP skills or a single test abnormality at 3 SD below the mean combined with reported symptoms that would correspond with such test deficits

Symptoms and risk factors	Reported listening difficulties and/or other symptoms described by the affected individual/their family/educational environment AND/OR presence of risk factors documented to be associated with or cause AP deficits	Symptoms and risk factors are summarized in Table 2

Non-verbal intelligence coefficient (IQ)	>80	It is acknowledged that findings of a borderline abnormal IQ may be due to testing limitations (e.g., instructions given in a noisy environment) rather than a true cognitive deficit *per se*

Ability to follow instructions in ideal conditions	Patient can understand and reliably follow instructions for the AP tests and reliably perform the pre-testing training	Criterion added to ensure that “non organic” cases, individuals who do not understand test instructions, not currently medicated ADHD patients, patients with uncontrolled psychiatrics symptoms, e.g., severe autism are not labeled as APD

### Future Research

It is essential to establish a scientifically valid evidence-based understanding concerning the direction of causality or the presence of a common causal pathology when APD is comorbid with cognitive deficits. The best way of approaching this matter at this time is to longitudinally evaluate individuals diagnosed with APD for cognitive skills by using large cohorts who are characterized in detail in terms of symptom description and other participant characteristics across different countries. These studies should also employ measures of brain activity with methods like electroencephalography, magnetoencephalography, and functional magnetic resonance imaging, which are frequently used in basic research studies. The already existing knowledge gained in such studies on the link between auditory cortex activity and performance in listening tasks in healthy subjects [e.g., Ref. (53, 54)] would then be translated to the clinical population. Comparing measures of central auditory processing in APD patients to the outcome of standard diagnostic procedures would contribute to differential diagnosis and identify predictors for long-term prognosis and management outcomes.

The benefit of such an approach with strong methodological quality will be to optimize diagnosis and arrive at a consensus of a standard diagnostic pathway that could be written down as a tree of decisions on whom to test, by which test means and when. In our joint effort to differentiate APD from other neurodevelopmental disorders or causes of listening difficulties we should not exclude the possibility of APD co-existing with other disorders or with multimodal deficits which may interact. Diagnosing APD when other disorders are present [ADHD, autism spectrum disorder, language impairment, dyslexia, learning disabilities (dementia in adults), emotional disorders] may benefit individuals by adding specific management or modifying existing instructions (i.e., FM systems, specific auditory training, individualized listening strategies/teacher based adaptations).

## Management

### Current Knowledge

The interventions (Table 2) should be as individualized as possible addressing (i) environmental modifications, (ii) use of FM systems (15), and (iii) systematic auditory training (55). Management needs to be multidisciplinary, and it is important that this is implemented in the educational environment for affected individuals who are still in education, in which case teacher-based adaptations and related strategies are of the utmost importance, alongside the previously mentioned interventions. Informal auditory training and compensatory, metacognitive, and metalinguistic strategies may also be of value (56, 57). Working memory deficits may be specifically trained as they may be closely linked to auditory perception, but they are a precondition for auditory-language processing, for example examined by dichotic listening tests. The client’s needs should be kept in mind before the multidisciplinary intervention planning. The audiologic results should be reflected in a multiprofessional background to figure out the appropriate and individualized management for each child under consideration of the child’s overall picture and problems in everyday life, the child’s individual resources, and that management interventions match well with the individual main problems in everyday life. It may benefit individuals with comorbid APD to provide them with individualized management even if complete recovery to normal auditory processing may not be a realistic expectation. Data showing short-term memory and general IQ improvement following auditory training and FM use should be further investigated as they are indicative of brain plasticity (9, 58, 59)—on condition that it is not a short-term improvement and the stability of the improvements is ensured. It is, however, of clinical interest that such outcomes are being reported in the difficult to test and complex mental health patient as well as in the neurological patient and the child diagnosed with APD.

**Table 2:** Symptoms, risk factors, and management of auditory processing disorder.

**Symptoms**	
Speech understanding difficulties	In background noise, acoustically challenging/complex acoustic environments, when speech quality is degraded
Speech discrimination difficulties	Difficulties to repeat or recall similar sounding words
Auditory memory/attention difficulties	Difficulties recalling instructions; difficulties concentrating in noise
Sound localization/streaming difficulties	Difficulties identifying the source of a sound; with separation of auditory foreground from auditory background
Relies on multisensory cues	E.g., seeking visual/facial cues to better understand
Hyperacusis	With or without a diagnosis of autism spectrum disorder
Disproportionate educational/cognitive/language difficulties	In the presence of normal audiometry and no other developmental disorders OR in the presence of normal audiometry and other diagnosed developmental disorders (specific language impairment; attention deficit disorder; autism; dyslexia) and (a) DESPITE implementation of appropriate interventions or (b) when other specialists or the educational environment seek further advice/assessment on management of the auditory aspect of this presentation

**Risk factors**	
Ear related	Intermittent middle ear pathologies, e.g., Chronic otitis with effusion (glue ear), recurrent upper respiratory tract infections
Brain related	Genetic or acquired neurological syndromes (e.g., brain tumors, traumatic brain injury, stroke, demyelination, etc.)
Development related	Attention deficit disorder; dyslexia; Specific language impairment; phonological disorder; autism spectrum disorders
Age related	Central presbyacusis

**Individualized management decided upon**	
Client considerations	Clinical characteristics, test results, overall needs, and preferences
Evidence	Best available evidence; of relevance to the particular client
Environment and resources	Availability of local resources; Client’s environment context; Related health/educational/workplace organizational context

**Key pillars of management**	
Listening strategies	Optimization of the listening environment (e.g., minimize noise); teacher-/speaker-based adaptations; other related strategies
Listening devices/systems	Frequency-modulated systems; sound field systems; hearing aid fitting with directional microphone to enhance SNR (signal-to-noise-ratio)
Auditory training	Formal and/or informal; chosen on the basis of patient’s AP test deficits/other symptoms and needs
Other means of management	Broader management of the client’s specific needs (e.g., reading deficiency; memory deficits; educational needs) by other agencies whenever needed and wherever possible

### Future Research

In order to implement cost-effective evidence-based APD management, there is a great need to identify specific psychoacoustic, clinical, or objective auditory markers (core measures) to guide appropriate specific management provision as opposed to using all three general approaches mentioned above. A European platform for collecting information on diagnostic approaches and APD intervention outcomes will be of great value to individuals experiencing listening, communicational, academic, and working/leisure difficulties. A European database would be used in this sense to check comparability of the prevalence of diagnosis, of symptomatology and of diagnostic test yield, usability of behavioral versus objective measures, and measure and compare management efficacy. The challenge in the European platform and database described would be to achieve verbal tests comparison across languages, instructions, and response mode uniformity with the addition of objective measures to compare across different languages and cultures.

## Author Contributions

This consensus paper is based on an idea by the first author (VI). A first draft was prepared by both corresponding author (D-EB) and first author (VI). All listed authors made substantial contributions to the conception of this European consensus and were involved in drafting the work and/or revising it critically for important intellectual content. Final approval of the version to be published was provided by all listed authors. All listed authors agree to be accountable for all aspects of the work in ensuring that questions related to the accuracy or integrity of any part of the work are appropriately investigated and resolved.

## Conflict of Interest Statement

The authors declare that the research was conducted in the absence of any commercial or financial relationships that could be construed as a potential conflict of interest. The reviewer [KI] and handling Editor declared their shared affiliation.

## Footnotes

^1^Other countries in Europe may have different ways of coding, e.g., in Germany, APD must be coded as F80.20 to get reimbursement from insurance companies.

## Abbreviations

ABR, Auditory Brainstem Responses; ANSD, Auditory Neuropathy Spectrum Disorder; APD, Auditory Processing Disorder; CAPD, Central Auditory Processing Disorder; HHL, Hidden Hearing Loss; HL, Hearing Loss; OAE, Otoacoustic Emissions; EEG, ElectroEncephaloGraphy; MEG, MagnetoEncephaloGraphy; fMRI, Functional Magnetic Resonance Imaging.
